# Association of Tissue mRNA and Serum Antigen Levels of Members of the Urokinase-Type Plasminogen Activator System with Clinical and Prognostic Parameters in Prostate Cancer

**DOI:** 10.1155/2014/972587

**Published:** 2014-04-29

**Authors:** Omar Al-Janabi, Helge Taubert, Andrea Lohse-Fischer, Michael Fröhner, Sven Wach, Robert Stöhr, Bastian Keck, Max Burger, Wolf Wieland, Kati Erdmann, Manfred P. Wirth, Bernd Wullich, Gustavo Baretton, Viktor Magdolen, Matthias Kotzsch, Susanne Füssel

**Affiliations:** ^1^Urologische Klinik, Universitätsklinikum Erlangen, Friedrich-Alexander Universität Erlangen-Nürnberg, 91052 Erlangen, Germany; ^2^Klinik für Urologie, Technische Universität Dresden, 01307 Dresden, Germany; ^3^Pathologisches Institut, Universitätsklinikum Erlangen, Friedrich-Alexander Universität Erlangen-Nürnberg, 91054 Erlangen, Germany; ^4^Department of Urology and Pediatric Urology, Julius-Maximilians-University, 97070 Würzburg, Germany; ^5^Clinic of Urology, Caritas-Hospital St. Josef, 93053 Regensburg, Germany; ^6^Institut für Pathologie, Technische Universität Dresden, 01307 Dresden, Germany; ^7^Department of Obstetrics and Gynecology, Technical University of Munich, 81675 Munich, Germany

## Abstract

The objective was to determine the mRNA expression and protein levels of uPA system components in tissue specimens and serum samples, respectively, from prostate cancer (PCa) patients and to assess their association with clinicopathological parameters and overall survival (OS). The mRNA expression levels of uPA, its receptor (uPAR), and its inhibitor type 1 (PAI-1) were analyzed in corresponding malignant and adjacent nonmalignant tissue specimens from 132 PCa patients by quantitative PCR. Preoperative serum samples from 81 PCa patients were analyzed for antigen levels of uPA system members by ELISA. RNA levels of uPA system components displayed significant correlations with each other in the tumor tissues. A significantly decreased uPA mRNA expression in PCa compared to the corresponding nonmalignant tissue was detected. High uPA mRNA level was significantly associated with a high Gleason score. Elevated concentration of soluble uPAR (suPAR) in serum was significantly associated with a poor OS of PCa patients (*P* = 0.022). PCa patients with high suPAR levels have a significantly higher risk of death (multivariate Cox's regression analysis; HR = 7.12, *P* = 0.027). The association of high suPAR levels with poor survival of PCa patients suggests a prognostic impact of suPAR levels in serum of cancer patients.

## 1. Introduction

Prostate cancer (PCa) is the second most frequently diagnosed malignancy and the sixth leading cause of cancer-related death in men worldwide [1]. Several biomarkers for PCa have been described either in tumor tissue or urine [2, 3]. However, further research and validation of these molecular markers for diagnostic and prognostic purposes in PCa patients are still necessary.

The urokinase-type plasminogen activator (uPA) system has been shown to play a key role in physiological and pathological pathways including cancer [4–7]. It consists mainly of uPA, the uPA receptor (uPAR), and the plasminogen activator inhibitor-1 (PAI-1). The uPA system is causally involved in multiple steps of cancer progression [6, 8, 9]. In addition to extracellular proteolysis, uPA in concert with uPAR and/or PAI-1 induces cell signaling pathways that affect tumor-associated processes such as angiogenesis, cell growth, cell adhesion and migration, chemotaxis, and cell survival [7, 8]. High expression levels of the uPA system members have been found to be associated with clinicopathological parameters and have an impact on disease prognosis in a variety of cancer types including prostate cancer [10–12].

In PCa, high uPA, uPAR, and/or PAI-1 protein expression in tumor tissue detected by immunohistochemistry (IHC) has been shown to be significantly associated with clinical prognostic parameters. Significant associations were observed between high immunoexpression of uPA and/or uPAR and a high Gleason score (GS), high tumor stage (pT), positive lymph node status, and incomplete tumor resection (R1) [13, 14]. Additionally, strong PAI-1 immunostaining was associated with high pT and R1 status [14]. Furthermore, biochemical recurrence-free survival of PCa patients with strong uPA, uPAR, and PAI-1 staining by IHC was significantly shorter than that of patients with weak staining [14]. Other research groups did not find significant associations between high uPA or PAI-1 immunoexpression and clinicopathological parameters [15]. However, the combined high uPA/PAI-1 immunoexpression was related to adverse pathological features, shorter overall survival (OS), and aggressive disease recurrence [15].

Only one study has been performed so far analyzing mRNA levels of the uPA system members in PCa tissues with regard to potential associations with clinicopathological data [16]. In that study, an increased mRNA expression of uPA and PAI-1 in PCa tissue compared to benign prostatic hyperplasia (BPH) tissues was detected. Furthermore, the mRNA expression of uPAR and PAI-1 was found to be associated with GS [16].

With regard to uPA, soluble uPAR (suPAR), and/or PAI-1 antigen levels in blood samples of PCa patients only few studies exist [17–20]. Elevated levels of either uPA or suPAR or elevated levels of both proteins in serum samples of PCa patients were found to be associated with shorter OS [17]. The pretreatment plasma levels of uPA and suPAR were mainly associated with early biochemical progression after radical prostatectomy [18]. In addition, by using specific immunoassay formats to detect individual, cleaved forms of suPAR in serum of PCa patients, the specificity of PCa detection may be improved [21]. Furthermore, using these assays to analyze suPAR (variant) levels in a cohort of 131 metastatic PCa patients it was demonstrated that high levels of all measured suPAR forms (including full-length suPAR) were significantly associated with a shorter OS [20].

In the present study of 132 PCa patients, the mRNA levels of uPA, uPAR, and PAI-1 were determined by quantitative PCR (qPCR) in tissue specimens. In addition, antigen concentrations of uPA system members were determined in preoperative serum samples from 81 of the 132 PCa patients and 36 patients with BPH by ELISA. Both the mRNA levels in tissue and the serum antigen concentrations were analyzed for correlations among these factors themselves as well as for their potential association with clinicopathological parameters and OS of the PCa patients.

## 2. Materials and Methods

### 2.1. Patients and Clinical Samples

The mRNA expression levels of uPA, uPAR, and PAI-1 were analyzed in corresponding malignant and adjacent nonmalignant prostate tissue samples of 132 nonselected PCa patients from two different urological centers. All of the patients in this study underwent radical prostatectomy at either the Department of Urology of the Technische Universität Dresden, Germany, or the Department of Urology of the University of Regensburg, Germany. The study was performed in compliance with the Helsinki Declaration. The use of prostatectomy tissues for research was approved by the Internal Review Boards from the Medical Faculties of the Universities of Dresden and Regensburg. All patients gave written informed consent.

The median age of the patients at surgery was 65 years (range 46–78 years). The median follow-up time after primary tumor resection was 83 months (range 14–150 months). During the follow-up period, 14 of the 132 patients (11%) died. The tumors were staged according to the UICC system and were graded according to the GS system.

From 81 out of the 132 PCa patients preoperatively collected serum samples were additionally available for the determination of antigen levels of uPA, suPAR, and PAI-1 in serum by ELISA. The median age in this patient subgroup was 65 years (range 48–77 years). The disease course of these patients was followed for a median period of 88 months (range 15–150 months) after surgery. During the follow-up period, seven of the 81 patients (8.6%) died. In addition, serum samples from 36 patients with BPH were available for analysis. The median age of BPH patients was 68 years (range 40–84 years). The relevant clinicopathological parameters of the PCa patient cohort are presented in Tables 1(a) and 1(b).

As there were only a few cases in our PCa cohort where cancer-specific death was documented, the overall survival defined as the time from radical prostatectomy to death or last contact (of any cause) or last contact of the PCa patients was used as follow-up end point.

### 2.2. Determination of mRNA Tissue Levels of uPA System Members by Quantitative PCR

Only tumor tissue samples with ≥70% cancer cells in the epithelial cell population and adjacent nonmalignant prostate tissue samples with less than 5% cancer cells were included. Total RNA was extracted from paired samples of malignant and nonmalignant tissues from each patient with the InviTrap Spin Tissue RNA Mini Kit (STRATEC Molecular GmbH, Berlin, Germany) according to the manufacturer's protocols. The cDNA synthesis was performed using Superscript II Reverse Transcriptase (Life Science/Invitrogen, Karlsruhe, Germany) according to the manufacturer's instructions.

Gene-specific primers and hybridization probes were used for the quantification of uPA, PAI-1, and uPAR mRNA levels (LC FastStart DNA Master Hybridization Probes; Roche Diagnostics, Mannheim, Germany) using Light Cycler technology (Roche) as previously described [22, 23]. Primer sequences, concentrations of primers and probes, and PCR conditions for amplification were as previously reported in detail ([24] and Supplemental Data Table 1 in Supplementary Material available online at http://dx.doi.org/10.1155/2014/972587). Six-log-range calibration curves were generated in each PCR run using PCR vessels coated with defined numbers of linearized plasmid molecules (10^1^–10^6^ molecules), carrying cDNA fragments of either uPA, PAI-1, uPAR, or TATA box binding protein (TBP) (AJ Roboscreen, Leipzig, Germany). The measurements were performed in independent duplicates using aliquots of the same cDNA dilutions to ensure comparable conditions.

The absolute quantification of gene expression was performed by the LightCycler software (LightCycler 480 SW 1.5, Roche). Relative mRNA expression levels of uPA, uPAR, and PAI-1 normalized to TBP were used for all statistical analyses.

### 2.3. Quantification of uPA System Member Protein Levels in Serum by ELISA

Serum samples obtained from 81 of the 132 PCa patients prior to radical prostatectomy and from 36 BPH patients were stored at −80°C until use. Antigen concentrations (ng/mL) of uPA, suPAR, and PAI-1 in serum samples were measured by specific ELISA formats (IMUBIND uPA ELISA #894, IMUBIND uPAR ELISA #893, and IMUBIND PAI-1 ELISA #821; Sekisui/American Diagnostica Inc., Stamford, CT, USA) according to the manufacturer's instructions.

### 2.4. Statistical Analysis

Correlations between continuous variables of the biological markers were calculated by Spearman's rank correlation test (*r*_*s*_). The relationships between the biological marker expression levels and clinicopathological parameters were assessed by Fisher's exact test. For this, the expression levels of uPA system members were separated by the median into low and high expression groups. The differences between mRNA expression levels of uPA system components in malignant and nonmalignant prostate tissues and between antigen concentrations in serum of PCa patients and BPH patients were estimated by Mann-Whitney *U* test. For survival analyses, OS, defined as the time from radical prostatectomy to death or last contact of the PCa patients, was used as follow-up end point. Statistical analyses of the association between the uPA system members and prognosis were performed using the Cox's proportional hazard regression model. The multivariate Cox's regression model was adjusted to relevant clinical prognostic factors: age, lymph node status, pT, and GS. The survival curves were estimated by Kaplan-Meier analysis (log-rank test). All calculations were performed using the SPSS 21.0 statistical package (SPSS-Science, Chicago, IL). The significance level was set at *α* = 0.05 and adjusted for multiple testing using Bonferroni correction.

## 3. Results

### 3.1. Relationship between mRNA Expression Levels of uPA System Members in Malignant and Nonmalignant Tissues from PCa Patients

All relative mRNA levels of the uPA system components displayed strong, significant, and positive correlations with each other. The relative mRNA expression levels of uPA correlated significantly with those of uPAR (*r*_*s*_ = 0.550; *P* < 0.001) and PAI-1 (*r*_*s*_ = 0.552; *P* < 0.001). In addition, a significant positive correlation was observed between the relative mRNA expression levels of uPAR and PAI-1 (*r*_*s*_ = 0.643; *P* < 0.001).

The comparison of mRNA expression of uPA system members in malignant tissue and adjacent nonmalignant tissue revealed that uPA mRNA expression values were significantly lower in the malignant tissue samples (*P* = 0.004). No significant differences occurred between malignant and adjacent nonmalignant PCa tissue for the expression of uPAR and PAI-1 (Supplemental Data Table 2A).

### 3.2. Associations between mRNA Expression Levels of uPA System Members in Tumor Tissue and Clinicopathological Parameters and Overall Survival of PCa Patients

**Figure 1:**
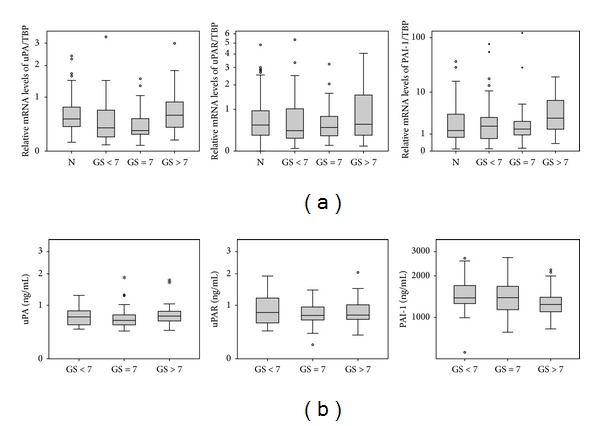
Levels of uPA system members in tumor tissues and serum in relation to Gleason score. The Gleason score was separated into three groups: GS < 7, GS = 7, and GS > 7; N: Nonmalignant tissue. (a) Relative transcript levels: mRNA values are given after normalization to TBP (TATA box binding protein) gene expression. (b) Serum protein levels: antigen concentrations are given in ng/mL.

The normalized relative mRNA expression values of uPA, uPAR, and PAI-1 in tumor tissue ranged from 0.08 to 11.48 (median 0.38), from 0.04 to 8.61 (median 0.47), and from 0.09 to 121.7 (median 1.75), respectively (Supplemental Data Table 2A). For statistical analyses, uPA, uPAR, and PAI-1 mRNA expression levels were stratified by the median values to separate PCa patients into groups with low and high tissue mRNA expression. The associations of mRNA expression levels with clinicopathological parameters of the PCa patients such as age, lymph node status, GS, and pT stage are summarized in Table 1(a). High uPA mRNA expression levels were significantly associated with high GS (*P* = 0.001; Table 1(a); Figure 1(a)). However, there was no association between the mRNA expression levels of the uPA system members and age, lymph node status, or tumor stage (Table 1(a)). No association between mRNA expression of uPA system members in tumor tissues and OS of the PCa patients was observed (data not shown).

### 3.3. Relationship between Antigen Levels of uPA System Members in Serum of PCa and BPH Patients

The concentrations of uPA, suPAR, and PAI-1 antigen were determined in preoperative serum samples of 81 PCa patients by ELISA. The median antigen concentrations were 0.68 ng/mL (range: 0.43–1.88), 0.76 ng/mL (range: 0.20–26.2), and 1347.9 ng/mL (range: 557.5–2714.0) for uPA, suPAR, and PAI-1, respectively (Supplemental Data Table 2B). The serum concentration of the uPA system members in PCa patients displayed only a rather weak but significant positive correlation in case of uPA versus suPAR (*r*_*s*_ = 0.382; *P* < 0.001). There were no correlations found between uPA and PAI-1 and suPAR and PAI-1 antigen levels in serum (*r*_*s*_ = 0.035; *P* = 0.756, and *r*_*s*_ = 0.133; *P* = 0.235, respectively). Likewise, in BPH patients a weak correlation was observed only between uPA and suPAR serum values (*r*_*s*_ = 0.407; *P* = 0.016, with Bonferroni correction *α* = 0.017).

In addition, there were no significant differences in serum antigen levels of uPA system members between PCa and BPH patients (Supplemental Data Table 2B). Furthermore, no correlation was detected between antigen levels and mRNA levels of uPA system components in serum and tissue of PCa patients, respectively (data not shown).

### 3.4. Associations between Antigen Levels of uPA System Members in Serum and Clinicopathological Parameters and Overall Survival of PCa Patients

After stratifying uPA, suPAR, and PAI-1 antigen values of PCa patients into low and high expression groups by the median values, potential associations with clinicopathological parameters of the PCa patients were assessed. The serum antigen levels showed no association with clinicopathological features of PCa patients (Table 1(b); Figure 1(b)).

**Figure 2:**
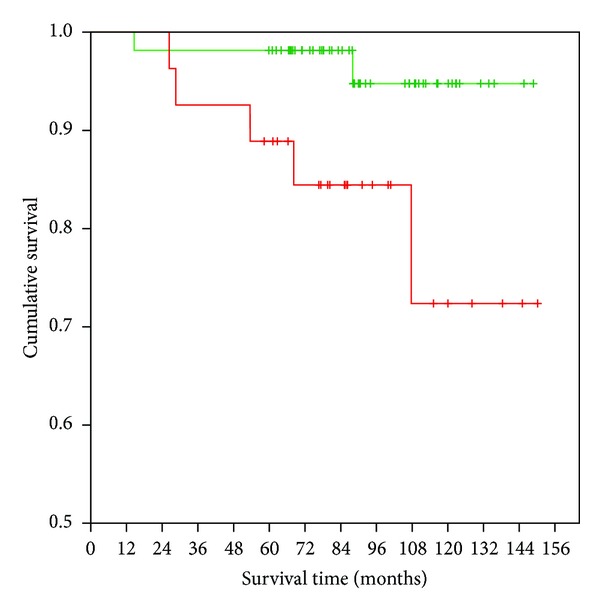
Association between uPAR protein concentration in serum samples and OS. Eighty-one patients were separated according to the 33% percentiles of serum uPAR protein concentration into subgroups of low/intermediate (green: 0–66% percentile; *n* = 54) and high uPAR (red: >66–100% percentile; *n* = 27) levels. OS was significantly shorter in the patient group with high uPAR levels compared with those patients with low/intermediate suPAR levels (*P* = 0.022; log-rank test).

For survival analyses we separated the PCa patients by the 33% percentiles (tertiles) into three groups with low, intermediate, or high serum levels of the uPA system components according to Almasi et al. [20]. Next we subsumed the patients with a low and intermediate serum level (0–66% percentile) and compared them to the group with high serum levels (>66–100% percentile) of the uPA system components. The antigen levels of uPA and PAI-1 in serum were not associated with prognosis of PCa patients neither in univariate nor in multivariate analysis (Table 2). Interestingly, the OS was significantly different for the suPAR antigen levels in Kaplan-Meier analysis (*P* = 0.022, log-rank test; Figure 2). The OS of PCa patients with low/intermediate preoperative suPAR serum levels averaged 144 months (95% CI: 138–150 months). The patients with high uPAR serum levels had a mean survival of 129 months (95% CI: 112–142 months). Moreover, elevated suPAR concentrations in serum were significantly associated with a distinctly higher risk of death of PCa patients in both univariate (HR = 5.48, 95%Cl: 1.06–28.29, *P* = 0.042) and multivariate (HR = 7.12, 95%Cl: 1.25–40.69, *P* = 0.027) Cox's regression analyses (adjusted to age, lymph node status, tumor stage, and Gleason score; Table 2). However, when the serum levels of uPA and PAI-1 were additionally included into the multivariate Cox's regression analysis, suPAR lost its statistical significance for OS (HR = 5.64, 95%Cl: 0.79–40.35; *P* = 0.085; Table 2). This may be caused by the significant positive correlation between uPA and suPAR serum levels.

## 4. Discussion

The aim of this study was to investigate tissue mRNA expression and serum antigen concentrations of uPA system members and to assess potential associations with clinicopathological and prognostic parameters in PCa patients.

The number of PCa studies aimed at assessing mRNA expression levels of uPA, uPAR, and PAI-1, particularly by qPCR, is very limited (Supplemental Data Table 3). A previous report has shown significantly higher mRNA expression levels of uPA and PAI-1, but not uPAR, in PCa tissue compared to benign and normal prostate tissue [16]. In the present study, no significant differences in mRNA expression levels of uPAR or PAI-1 were found between malignant and adjacent nonmalignant tissue of PCa patients. However, the expression levels of uPA were significantly lower in PCa tissue compared to the corresponding nonmalignant tissue specimens. One explanation for these discrepancies may be the different origin of nonmalignant tissue samples used in the two studies. In contrast to our study, where adjacent nonmalignant tissue was investigated, Riddick et al. compared mRNA expression of uPA system components in malignant tissues (44 PCa) with either benign prostatic tissue (23 BPH patients) or normal prostatic tissues from patients treated for bladder cancer [16]. In general, the cellular composition and expression of uPA components in adjacent nonmalignant tissue of PCa patients, as used in our study, may be different from that of benign prostate tissue of BPH patients. Furthermore, methodological differences in qPCR techniques (different primers and reference genes) or the composition of patient cohorts (different GS subgroups) may further explain discrepancies in marker detection.

The cellular distribution of uPA system components has been investigated mainly in PCa tissue in comparison to BPH specimens [25, 26]. uPA, uPAR, and PAI-1 were detected in the majority of PCa and BPH tissues by both* in situ* RNA hybridization and IHC. Marker expression was mainly observed in different types of stromal and immune cells such as fibroblasts, macrophages, or neutrophils, but not in cancer cells or other epithelial cells [25]. Conversely, by analyzing uPA and uPAR expression in PCa and BPH tissues using the same techniques, Gavrilov et al. found expression signals predominantly in adenocarcinoma cells of high grade PCa. The stromal cells within tumor tissue and in BPH tissues were mostly negative for both markers [26]. This controversy regarding the staining patterns of uPA system components in PCa or BPH tissues may be related to methodological differences concerning mRNA hybridization or the specificity of antibodies used for IHC [25]. No comparative study using paired malignant and adjacent nonmalignant tissues from PCa patients has been performed so far.

Significant correlations between the mRNA expression levels of uPA, uPAR, and PAI-1 were detected in our study. In agreement with these results, strong positive correlations between mRNA values of uPA system components have been observed in other types of cancer such as breast cancer, pancreatic adenocarcinoma, and soft tissue sarcomas [23, 24, 27–30] but were not reported in PCa yet. Besides the established fact of various protein/protein interactions of the members of the uPA system, the genes encoding them are likely regulated in a concerted manner in tumor growth and metastasis, for example, by the hypoxia-dependent transcription factor HIF1*α* as shown for uPAR und PAI-1 [31] and by CD44 as reported for uPA and uPAR [32].

A significant association between elevated mRNA levels of uPA (Table 1(a)) and a higher GS was detected in our study. However, our data showed no significant association between mRNA levels of uPA system components and other clinicopathological parameters such as tumor stage and lymph node status of PCa patients.

So far, protein levels of uPA, uPAR, and/or PAI-1 in tumor tissue from PCa patients were assessed by IHC studies ([13–15]; Supplemental Data Table 3). However, controversial results were obtained regarding the association of uPA system components with prognostically relevant clinicopathological parameters [13–15]. Whereas in some studies [13, 14] high tissue levels of uPA, uPAR, and/or PAI-1 protein have been shown to be significantly associated with clinical prognostic parameters (GS, tumor stage, lymph node status, and tumor resection status), others did not find significant associations between high immunoexpression of uPA system components and clinicopathological parameters [15]. The discrepancies between these studies on protein immunostaining of the uPA system components, as well the partial incoherency to our mRNA-based study, indicate that there might be no clear relation to GS, tumor stage, and lymph node status in PCa patients.

In addition, we were interested whether the tissue mRNA expression of the uPA system members was associated with OS of PCa patients. However, no association between tissue mRNA expression levels of the uPA system members and OS of PCa patients was detected. Our results are consistent with other studies that found no relationship between uPA, uPAR, and/or PAI-1 mRNA expression and patients' survival in breast cancer, pancreatic cancer, and soft tissue sarcoma [24, 29, 33]. Conversely, other studies have demonstrated that mRNA expression levels of uPA system components were linked to the prognosis of patients with various types of cancer such as breast, pancreatic, gastric, or colorectal cancer [27, 28, 30, 34–40].

In studies aimed at the analysis of the antigen concentrations of the uPA system members in blood, significant associations of high uPA and/or suPAR levels with nonorgan-confined, advanced disease [17, 20] or with a high GS [18] of PCa patients, were observed (Supplemental Data Table 3). In the present study, we found no significant associations between serum levels of uPA system components and clinicopathological parameters of PCa patients. Nevertheless, elevated suPAR serum levels were significantly associated with a shorter OS of PCa patients (log-rank test: *P* = 0.022). Furthermore, in multivariate Cox's regression analyses, high suPAR levels in serum were significantly associated with a 7-fold higher risk of death (of any cause) of PCa patients. However, when the serum levels of all three uPA system members were included simultaneously in the multivariate Cox's regression analysis for OS, suPAR level did not remain a significant prognostic marker. This may be due to the significant correlation between serum levels of uPA and suPAR in the present PCa cohort. Our results are consistent with previous reports that found a significant association between high levels of suPAR in blood and poor prognosis of PCa patients [17, 18, 20]. However, the design of these studies differed from that of the present study. Miyake et al. measured suPAR (and uPA) in serum from a cohort of PCa patients composed of a high proportion of patients with distant metastasis [17]. Significantly shorter OS of PCa patients with elevated levels of suPAR and/or uPA, but not of the single markers, was observed compared to patients with low levels of both. In our study, we did not find an additive effect on prognosis, when assessing combinations of uPA system members (data not shown). Recently, Almasi et al. demonstrated that elevated pretreatment levels of full-length suPAR and cleaved forms of suPAR in serum were significantly associated with shorter OS in a cohort of 131 patients with metastatic PCa [20]. Unlike the studies mentioned above, the PCa patient cohort in our retrospective study consisted of nonselected patients with primary, clinically localized PCa with a very low rate of distant metastasis.

In a study on uPAR and uPA levels in plasma of PCa patients with clinically localized PCa, Shariat et al. reported that high preoperative suPAR (and uPA) values were associated with patients' prognosis. However, in contrast to our study, early biochemical progression as estimated by increase in PSA values was used as end point for the prognostic evaluation [18]. In our study, we present strong evidence for the association of high suPAR serum levels with shorter OS in a well-defined patient cohort with clinically localized prostate cancer. Therefore, soluble uPAR measured in serum or plasma of PCa patients is suggested to be a potential prognostic marker for PCa patients. Similarly, high serum levels of suPAR were reported to be associated with worse prognosis in several studies on other types of cancer [for review see [41, 42]].

All in all, the present status of results concerning associations of uPA system members at the mRNA and protein level in tumor tissue with clinicopathological parameters in PCa strongly implies a standardized, prospective study of the uPA system in PCa tissues. The association of high suPAR levels with poor survival of patients with PCa or other cancers indicates a prognostic impact of suPAR antigen levels measured in blood.

## Supplementary Material

Supplementary Data: The Supplemental Data comprise three tables (Table 1, Table 2A, Table 2B and Table 3).
Supplementary Data Table 1: provides the amplification primers and detection probes
Supplementary Data Table 2A: describes the median mRNA expression values and ranges of the uPA system members in malignant tissue and corresponding nonmalignant tissue of PCa patients.
Supplementary Data Table 2B: presents the median antigen concentrations and ranges of the uPA system members in serum of PCa and BPH patients.
Supplementary Data Table 3: gives an overview of the literature and the results of this study for associations of tissue RNA or protein levels of uPA gene family members with clinicopathological parameters in prostate cancer.

## Figures and Tables

**Table:** (a)

Clinicopathological parameters	Number of cases	uPA low/high	uPAR low/high	PAI-1 low/high
	132			
Age		*P* = 0.602	*P* = 1.000	*P* = 0.602
<65 yrs	66	35/31	33/33	35/31
≥65 yrs	66	31/35	33/33	31/35
Lymph node status*		*P* = 0.796	*P* = 0.795	*P* = 0.434
pN0	107	56/51	57/50	58/49
pN1	17	8/9	8/9	7/10
Tumor stage		*P* = 0.081	*P* = 0.296	*P* = 0.163
pT2	69	40/29	38/31	39/30
pT3 + 4	63	26/37	28/35	27/36
Gleason score		**P** = 0.001	*P* = 0.199	*P* = 0.007
GS < 7	35	19/16	21/14	17/18
GS = 7	56	36/20	28/28	36/20
GS > 7	41	11/30	17/24	13/28

Statistical analysis was performed using Fisher's exact test. Significant value is marked in bold. The Bonferroni-adjusted threshold for significance is at *α* = 0.0042.

*Number of patients *n* = 124.

**Table:** (b)

Clinicopathological parameters	Number of cases	uPA low/high	uPAR low/high	PAI-1 low/high
	81			
Age		*P* = 0.025	*P* = 0.377	*P* = 0.508
<65 yrs	40	25/14	23/17	22/18
≥65 yrs	41	15/26	19/22	19/22
Lymph node status*		*P* = 0.311	*P* = 0.5185	*P* = 0.311
pN0	69	36/33	36/33	33/36
pN1	10	3/7	4/6	7/3
Tumor stage		*P* = 0.263	*P* = 0.508	*P* = 0.825
pT2	43	24/19	24/19	21/22
pT3 + 4	38	16/22	18/20	19/19
Gleason score		*P* = 0.159	*P* = 0.838	*P* = 0.429
GS < 7	18	7/11	9/9	8/10
GS = 7	42	25/17	23/19	19/23
GS > 7	21	8/13	10/11	13/8

Statistical analysis was performed using Fisher's exact test. The Bonferroni-adjusted threshold for significance is at *α* = 0.0042.

*Number of patients *n* = 79.

**Table 2:** Univariate and multivariate Cox's regression analysis of the association of uPA, PAI-1, and uPAR serum levels in PCa patients with OS (*n* = 81).

Parameters	Number of cases	Univariate analysis	*P*	Multivariate analysis^1^	*P*	Multivariate analysis^2^	*P*
		HR (95% CI)		HR (95% CI)		HR (95% CI)	
	81						
uPA serum*							
Low/intermediate	54	1		1		1.15 (0.12–6.42)	0.890
High	27	3.34 (0.73–15.20)	0.119	2.49 (0.51–12.25)	0.261	1	
PAI-1 serum*							
Low/intermediate	54	1		1		1	
High	27	2.53 (0.56–11.30)	0.226	4.94 (0.85–28.76)	0.076	3.06 (0.53–17.55)	0.209
uPAR serum*							
Low/intermediate	54	1		1		1	
High	27	**5.48 (1.06–28.29)**	**0.042**	**7.12 (1.25–40.69)**	**0.027**	5.64 (0.79–40.35)	0.085

HR: hazard ratio (95% confidence interval) of the univariate and multivariate Cox's regression hazard analysis.

Multivariate analysis was adjusted to relevant clinicopathological parameters: ^1^age, lymph node status, tumor stage, and Gleason score.

^2^Age, lymph node status, tumor stage, and Gleason score and with all three uPA components covariates added at once.

*Serum antigen levels of uPA system components (ng/mL) were divided into low, intermediate, and high levels by the 33% percentiles (tertiles); low/intermediate: 0–66% percentile; high: >66–100% percentile.
