# Supplementary material for: Association of Tissue mRNA and Serum Antigen Levels of Members of the Urokinase-Type Plasminogen Activator System with Clinical and Prognostic Parameters in Prostate Cancer
Source: Biomed Res Int. 2014 Apr 29;2014:972587. doi: 10.1155/2014/972587 (PMC4022202; doi:10.1155/2014/972587)
Supplement: Supplementary file 1 — Supplementary Data: The Supplemental Data comprise three tables (Table 1, Table 2A, Table 2B and Table 3). Supplementary Data Table 1: provides the amplification primers and detection probes Supplementary Data Table 2A: describes the median mRNA expression values and ranges of the uPA system members in malignant tissue and corresponding nonmalignant tissue of PCa patients. Supplementary Data Table 2B: presents the median antigen concentrations and ranges of the uPA system members in serum of PCa and BPH patients. Supplementary Data Table 3: gives an overview of the literature and the results of this study for associations of tissue RNA or protein levels of uPA gene family members with clinicopathological parameters in prostate cancer. [file 972587.f1.pdf]

Supplemental Data Table 1A. Relative mRNA expression levels of the uPA system members in tissue of PCa patients

	No. of patients	Median[1]	Range
uPA		***P* = 0.004**	
Malignant tissue	132	0.38	0.08 – 11.5
Non- malignant tissue	125	0.51	0.12 – 2.39
uPAR		*P* = 0.604	
Malignant tissue	132	0.47	0.04 – 8.61
Non- malignant tissue	125	0.54	0.00 - 25.8
PAI-1		*P* = 0.261	
Malignant tissue	132	1.75	0.09 – 121.7
Non- malignant tissue	125	1.31	0.09 – 37.4
Ratio uPA/PAI-1		*P* = 0.036*	
Malignant tissue	132	0.21	0.01 – 3.81
Non- malignant tissue	125	0.33	0.03 – 3.15

mRNA expression levels of the *uPA* system members are normalized to *TATA box binding protein* (*TBP*) gene expression. The Bonferroni-adjusted threshold for significance is at α=0.0125. [1] Mann-Whitney test, * Wilcoxon rank test.

Supplemental Data Table 1B. Antigen concentrations of the uPA system members in serum of PCa and BPH patients

	No. of patients	Median[1]	Range
**uPA**		*P* = 0.303	
PCa serum	81	0.68	0.43 – 1.88
BPH serum	36	0.68	0.51 – 2.21
**uPAR**		*P* = 0.692	
PCa serum	81	0,76	0,20 – 26,2
BPH serum	36	0.79	0.05 – 1.46
**PAI-1**		*P* = 0.310	
PCa serum	81	1347,9	557,5 – 2714,0
BPH serum	36	1385.5	940.9 – 5930.2
**PSA**		***P* < 0.001**	
PCa serum	81	9.21	1.29 – 51.9
BPH serum	36	1.78	0.22 – 17.5

Antigen concentration in serum is given in ng/ml; The Bonferroni-adjusted threshold for significance is at α=0.0125. [1] Mann-Whitney-Test.

Supplemental Data Table 2. Overview of literature including the present study: Association of tissue RNA or protein levels of uPA gene family members with clinicopathological parameters in prostate cancer.

Factor	Number of patients	Method	Significant association / Remarks	Reference
**Tissue RNA**				
uPA	13 locally recurrent hormone refractory PCa<br>PCa cell lines: PC-3, LNCaP, DU145	qPCR;<br>Southern hybridization	*uPA* mRNA with *uPA* gene amplification in refractory PCa<br>PC-3 with gene amplification more sensitive to uPA inhibitor	Helenius *et al.* 2001
uPA, *uPAR*	25 high grade PCa	*in situ* hybridization; IHC	*uPA* and *uPAR* expression in PCa and BPH; *uPA* and *uPAR* expression predominantly in adenocarcinoma cells of high grade PCa; Stromal cells were mostly negative for both *uPA* & *uPAR*	Gavrilov *et al.* 2001
uPA, *uPAR*, *PAI-1*	44 PCa, 23 BPH	qPCR	Increased expression of uPA and PAI-1 in malignant compared with non-malignant prostate tissue; uPAR and PAI-1 with higher GS	Riddick *et al.* 2005
uPAR, *PAI-1*	stromal cell cultures		uPAR and PAI-1 were primarily expressed by stromal cells	Riddick *et al.* 2005
uPA, *uPAR*, *PAI-1*	16 PCa , 9 BPH	*in situ* hybridization; IHC	*uPA*, *uPAR* and *PAI-1* expression in both PCa and BPH; *uPA* and *uPAR* expression predominantly in macrophages, *PAI-1* expression mostly in scattered fibroblast-like cells, few in macrophages; No association between *uPA*, *uPAR* or *PAI-1* expression and GS	Usher *et al.* 2005
uPA, *uPAR*, *PAI-1*	132 PCa	qPCR	Strong correlation between expression levels of all three *uPA* system members; *uPA* with higher GS	Present study
**Tissue Protein**				
uPA, uPAR	120 clinically localized PCa	IHC	uPA and uPAR with GS, tumor stage, surgical margin status; uPA with lymph node status	Cozzi et al. 2006
uPA, PAI-1, uPAR	230 clinically localized PCa	IHC	Combined uPA/PAI-1 values with extraprostatic extension and seminal vesicle involvement; uPA and PAI-1 with biochemical RFS and aggressive disease recurrence; combined uPA/PAI-1 values independent predictor	Gupta et al. 2008

			for biochemical RFS and aggressive disease	
uPA, PAI-1, uPAR	153 PCa with organ-confined disease	IHC	uPA and uPAR with tumor stage, GS, lymphatic invasion, surgical margin status, and lymph node metastasis; PAI-1 with tumor stage and surgical margin status; uPA, uPAR and PAI-1 with shorter biochemical RFS; uPA independent predictor for biochemical RFS	Kumano et al. 2009
**Blood-based Protein**				
uPA, suPAR	72 PCa (65% metastatic), 62 BPH, 54 healthy donors	EIA (Serum)	Increased uPA and uPAR levels in PCa compared to BPH patients; uPA and uPAR with tumor stage and metastasis; Either uPA or uPAR or both with OS	Miyake et al. 1999
uPA, suPAR	429 clinically localized PCa, 19 PCa with lymph node metastasis, 10 PCa with bone metastases, 44 healthy donors	EIA (Plasma)	uPA and uPAR with tumor stage, GS, lymph node metastasis and lymphatic invasion; uPA and uPAR with features of biologically aggressive PCa, disease progression after radical prostatectomy and metastasis; uPA and uPAR in PC patients with bone metastasis>PCa with lymph node metastasis> localized PCa> healthy men	Shariat et al. 2007
uPA, suPAR, PAI-1	423 clinically localized PCa	EIA (Serum)	uPA and uPAR with biochemical RFS	Shariat et al. 2008
suPAR	131 metastatic PCa	TR-FIA (Serum)	Each of the suPAR forms with shorter OS	Almasi et al. 2011
uPA, suPAR, PAI-1	81 PCa, 36 BPH	EIA (Serum)	uPAR with shorter OS	Present study

Table was modified according to Schmitt et al. 2010; if not indicated otherwise, high marker levels were associated with clinical parameters;

Abbreviations: PCa: Prostate Cancer; BPH: benign prostatic hyperplasia; qPCR: quantitative PCR; EIA: Enzyme-linked immunosorbent assay; TR-FIA: Time-resolved fluorescence immunoassay; IHC: immunohistochemistry; GS: Gleason score; OS: Overall survival; RFS: recurrence-free survival
